# Association of Human Papillomavirus Infection with Tonsillar Cancers: A Systematic Review

**DOI:** 10.1007/s12070-023-04140-2

**Published:** 2023-08-29

**Authors:** Sneha Sethi, Alana Shahin, Intisar Nuha Abd Rahim

**Affiliations:** 1 Australian Research Center for Population Oral Health, Adelaide Dental School, Faculty of Health and Medical Sciences, The University of Adelaide, Adelaide, SA 5000 Australia; 2 Adelaide Medical School, Faculty of Health and Medical Sciences, The University of Adelaide, Adelaide, SA Australia

**Keywords:** HPV, Tonsillar cancer, Systematic reviews, Oropharyngeal health

## Abstract

Vaccinations have shown a decrease in human papillomavirus (HPV) infection-related cervical cancer in women, but there has been a sharp rise in the HPV infection-related oropharyngeal cancer cases over the past few decades. Recent studies have suggested the association of HPV infections with tonsillar cancers as well and suggestions regarding preventive tonsillectomies in order to achieve a decrease in HPV infection-related oropharyngeal or tonsillar cancer have arisen. However, there is limited cumulative evidence validated at a global level to support the endorsement of this strategy. This research revolves around the concept of burden of tonsillar carcinomas due to oropharyngeal HPV infection. Thus, a systematic review and meta-analysis of existing studies was undertaken to estimate the pooled prevalence of tonsillar cancer associated with oropharyngeal HPV infection. Published articles on tonsillar cancer with and without HPV infection from PubMed, Embase, Scopus and Web of Science were systematically searched from inception until 23 December 2021. A random-effects model was used to estimate the pooled prevalence forest plots. The systematic review revealed that 50% of the reported cases of tonsillar cancer had an oropharyngeal HPV infection, questioning the preventive nature of an early tonsillectomy which is essentially an invasive surgical procedure. Large heterogeneity was reported in the included studies, and there was insufficient data for sub-group analysis. Future research and representative studies are required to thoroughly explore the correlation between HPV infection and tonsillar cancer.

**Supplementary Information:**

The online version contains supplementary material available at 10.1007/s12070-023-04140-2.

## Background

Human papillomavirus (HPV) infection is one of the most common sexually transmitted infections, prevalent across the globe, with its incidence increasing drastically since the 1970s [1–3]. There are currently more than 150 recognised genotypes of HPV, which are a diverse heterogeneous group of viruses, with circular, double-stranded, non-enveloped DNA, with the ability to grow within transitional stratified squamous epithelium of skin and mucous membranes [2, 3]. The common basis of categorising and distinguishing the different types of HPV is based on the homologous L1 nucleotide sequence in the virus’s DNA [3]. There are 30 HPV types grouped under the umbrella of genus alpha-papillomavirus, also called ‘mucosal HPV types’ as they have been found to infect oropharyngeal and genital mucosal tracts [3]. Roughly, 14 subtypes of HPV are ‘high-risk’ (hr-HPV) known to initiate carcinogenic cellular changes, thus making genital and oropharyngeal mucosal tracts vulnerable to cancer associated with persistent HPV infections [2–4].

Cervical cancer is the fourth most common cancer in women with approximately 290 million women currently infected with an HPV infection corresponding to 570,000 cervical cancer cases [4, 5]. Almost all (99%) cases of cervical cancer are associated with hr-HPV (specifically HPV 16 and /or 18), with an associated mortality of 311,000 cases, globally [3–5]. The association of HPV infections with cervical cancer was first reported in 1983 by Zur Hausen et al. [3, 6]. Around the same time the first evidence between oropharyngeal carcinogenesis and HPV infection was reported by Syrjänen’s et al. [3, 7]. Furthermore, head and neck squamous cell carcinoma (HNSCC) accounts for nearly 3–5% of all cancers and is the sixth most common cancer, effecting the lip, oropharyngeal, hypopharynx, larynx, sino-nasal tract and nasopharynx [3]. Recently, oropharyngeal carcinoma has seen a drastic increase in prevalence compared to other sites like the lip and tongue [3, 8]. Due to particular characteristics of the epithelial lining in tonsillar crypts, oropharyngeal HPV infections are most commonly associated with tonsils and the oropharynx [3]. Oropharyngeal cancers occur in specific regions of the throat, specifically the posterior third of the tongue, posterior walls of the oropharynx and the tonsils [3].

The beginning of the twenty-first century saw campaigns across various countries aiming to reduce tobacco smoking rates, in order to eventually decrease the incidence of tobacco-related cancer burden [8]. However, during this time, the prevalence of oropharyngeal cancers also rose, acknowledging the association with oropharyngeal HPV infection [7, 8]. OPSCCs associated with oropharyngeal HPV infections increased from 20% to over 70% in USA, with similar statistics observed in other developed countries as well [8]. The incidence has since continued to rise and is now a recognised significant global public health concern. The American Centre of Disease Control and Prevention (CDC) in 2012, reported that oropharyngeal cancer is the most commonly diagnosed cancer associated with HPV infections, with its incidence now surpassing uterine cervical cancer.

Whilst there is a marked associated between oropharyngeal cancer, tobacco and alcohol, the drastic rise in global cases is largely attributed to the complex correlations with persistent oropharyngeal HPV infections [3, 8]. A study based in Denmark has reported that, approximately 77% of tonsil carcinomas reported between 2000 and 2010 were related to oropharyngeal infection with hr-HPV [9, 10]. Transmission of HPV infections is primarily via sexual contact, and oropharyngeal HPV infections can be explained by unsafe oral-sexual behaviours, like number of oral sexual partners, unprotected oral sexual encounters [8]. Hypothetically, elevated incidence in men could be due to sexual (or oro-sexual) contact with a female/male having a hr-HPV genital infection [8]. It has also been reported that HPV-associated oropharyngeal carcinomas disproportionately affect men, which is about three to five times higher as compared to women [3, 4, 8]. Increased prevalence amongst the younger demographic, with lesser alcohol and smoking exposure, can be explained by unsafe sexual behaviours leading to an increased oropharyngeal exposure of HPV infected anogenital sites [2, 8].

Recent studies have explored a correlation between the incidental surgical removal of the tonsils at a young age with an observed subsidiary decline in the associated incidence of oropharyngeal or tonsillar cancers [1, 11]. Tonsillectomies are a commonly observed and practised procedure, especially in children [12]. A study group in Sweden followed a cohort of participants with and without tonsillectomies, to explore this correlation and reported to have observed a reduced risk of tonsillar cancer [1]. However, a large proportion of the research is yet to be validated, and is mostly circumstantial lacking solid scientific evidence. Some studies have also found traces of HPV-16 even though the tonsils were removed [12–14]. Cancerous changes cannot manifest if an organ tissue no longer exists, the question arises whether recommending tonsillectomies are justified as a means of “carcinoma prophylaxis” to avoid tonsillar and/or oropharyngeal cancers.

The aim of this study was to determine the correlation between the presence of HPV and the incidence of tonsillar cancer. It was hypothesized that there is a positive correlation between HPV infections in transitional epithelium of tonsils leading to increased incidence of tonsillar cancer.

## Methods

This systematic review is registered in PROSPERO (CRD42022306602) and was performed following the Preferred Reporting Items for Systematic Reviews and Meta-Analyses statement [15].

### Data Sources and Searches

A structured literature search (Online Appendix 1) was performed using the search engines PubMed, Scopus, Embase and Web of Science until December 2021. The search strategy for PubMed included the operators ‘OR’ and ‘AND’, in combination with subject terms (‘MeSH Terms’) and free text terms (‘Text Word’). These terms were collated to compose the following search: (“HPV”[Text Word] OR “papillomavirus”[MeSH Terms] OR “human papillomavirus”[Text Word] OR “human papillomavirus 16”[MeSH Terms] OR “human papillomavirus 16”[Text Word] OR “human papillomavirus 18”[MeSH Terms] OR “human papillomavirus 18”[Text Word] OR “papillomavirus”[Text Word]) AND (“Tonsils”[Text Word] OR “tonsil*”[All Fields]) AND (“cancer”[Text Word] OR “carcinoma”[Text Word] OR “Malignant”[Text Word]). The reference lists of all relevant papers were manually checked to identify any additional applicable studies.

### Study Selection

**Fig. 1:**
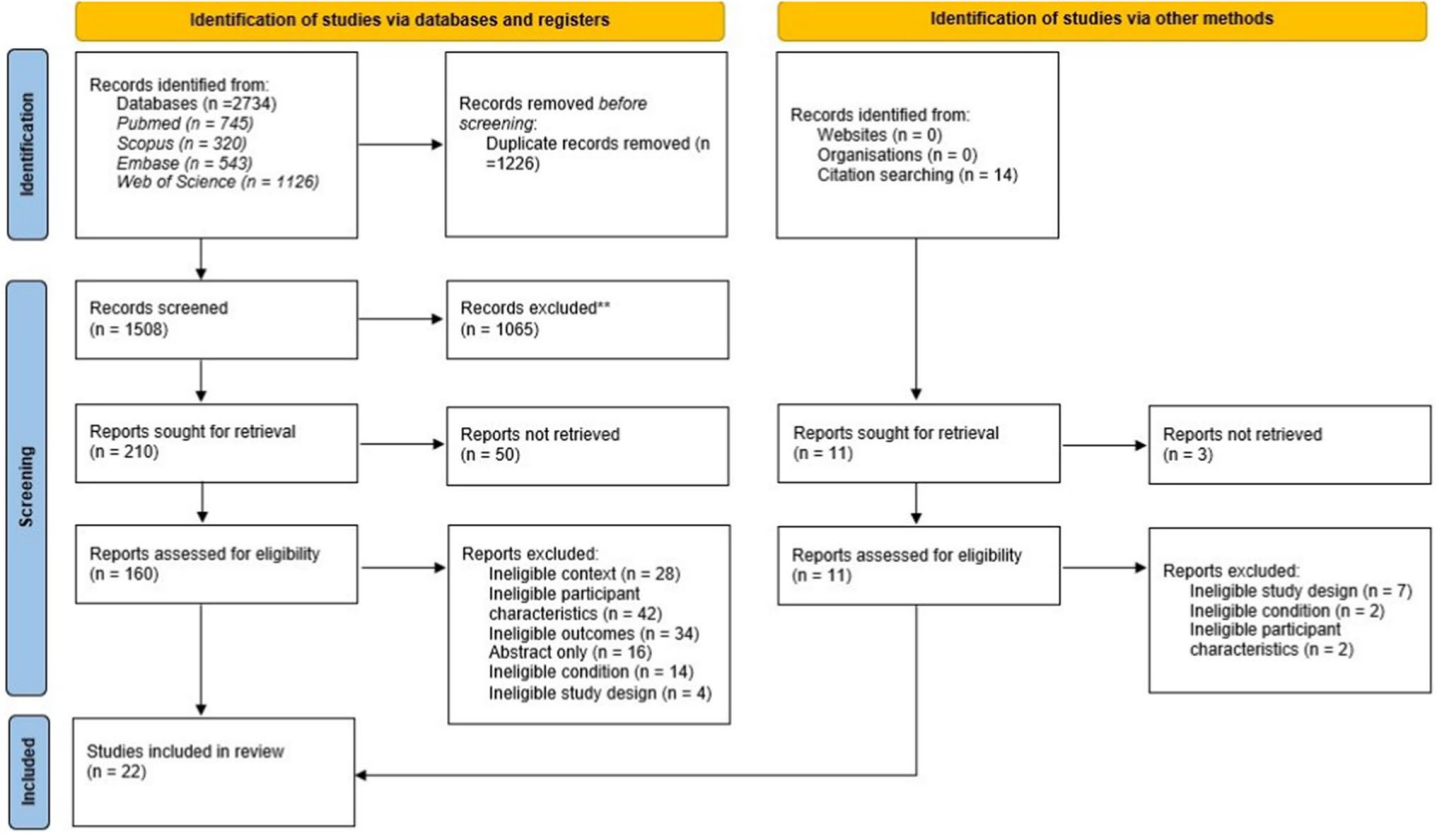
PRISMA 2020 flow diagram for this systematic review which included searches of databases, registers and other sources

The inclusion criteria for studies to be included were predefined (Fig. 1). All observational studies (cross-sectional, cohort and case–control) were included, excluding studies presenting secondary evidence, like systematic reviews. Case reports, case studies and interventional studies (eg clinical trials) were excluded. Titles and abstracts were retrieved and analysed by two independent reviewers (A.S. and I.R.). Disagreements were resolved by a discussion consensus involving a third reviewer (SS). Full texts of all selected studies were collected and the aforementioned inclusion criteria were applied. Duplicates were removed and studies which did not include HPV infection associated tonsillar cancer, not available in English or had no full-text available were excluded.

### Data Extraction and Quality Assessment

Data was extracted from the final curated list of relevant studies, and collated into an Excel spreadsheet. The extracted and recorded data included: first author, country and year of publication, number of total cancer cases, number of tonsillar cancer cases, and number of HPV positive tonsillar cancer patients [16]. Two reviewers then independently performed screening on the basis of definite inclusion and exclusion criteria. Additionally, the reviewers appraised the studies for quality and risk bias, using the Joanna Briggs Institute (JBI) appraisal tools. This was measured by a series of nine: yes, no, unclear, not applicable questions (Online Appendix 3) evaluating the quality of the sample, methodology, analysis and bias of the studies. A third reviewer (S.S) evaluated the outcomes and summaries of the appraisal results, which were then tabulated.

### Statistical Analysis

The aim of this study was to calculate the prevalence of tonsillar cancer within a group of head and neck cancer lesions, and additionally evaluate the proportion of cases associated with an oropharyngeal HPV infection. Studies which specified all of the three parameters (cancer status, site (tonsils) and HPV infection status) were included. After extracting the data, a random-effects model was used to estimate the pooled prevalence and 95% confidence intervals. I^2^ statistic was calculated to determine the heterogeneity between the included studies. An I^2^ statistic greater than 50% demonstrated a high level of heterogeneity between studies [16].

## Results

### Study and Patient Characteristics

A total of 2734 studies were accrued from PubMed and Web of Science, and collated into a reference list on EndNote. After removal of duplicates, 1508 were initially screened of their titles for relevance to the study, with 1065 excluded after this process, and 210 excluded after the reviewing of abstracts. Following the aforementioned exclusion and inclusion criteria, 22 studies were included for analysis, as illustrated in the flow chart [15] of Fig. 1.

Geographically, these included studies were from the six continents, where majority of studies [9, 17–29] involved Europe, three studies involved varying regions of China [30–32], and one study from each Australia [33], North and South America [34, 35], Africa [36] and the Middle East [37]. These details, and further characteristics of these studies are listed in Table 1.

**Table 1:** Summary of the characteristics of studies

Year	Author	Country	Total cases	Tonsillar cancer	HPV (+) tonsillar cancer
2006	Sacramento et al.	Brazil	173	39	3
2008	Romanitan et al.	Greece	115	31	12
2009	Nasman et al.	Sweden	253	162	128
2010	Blomberg et al.	Denmark	26,474	2226	1296
2010	Hong et al.	Australia	300	188	83
2012	Huang et al.	China	66	27	7
2014	Nordfors et al.	Sweden	76	29	18
2014	Rodrigo et al.	Spain	248	140	6
2015	Blumberg et al.	Mozambique	51	7	0
2015	Henneman et al.	Netherlands	146	78	37
2015	Melchers et a.l	Netherlands	193	120	31
2016	Lam et al.	China	207	124	36
2016	Schache et al.	UK*	1519	854	528
2017	Carlander et al.	Denmark	700	487	299
2017	Fossum et al.	SE*-Norway	166	89	71
2018	Carpen et al.	Finland	105	65	38
2019	Bozinovic et al.	Croatia	99	53	23
2019	Haeggblom et al.	Sweden	295	196	147
2020	Alexiev et al.	USA*	332	301	288
2020	Crotty et al.	Ireland	30	16	14
2020	Maroun et al.	Middle East#	34	14	2
2020	Xu et al.	China	170	109	70

*UK United Kingdom, *SE* South Eastern, *USA* United States of America

^#^Included—Lebanon, Syria, Palestinian Territories, Jordan, Iran

### Study Quality

**Fig. 2:**
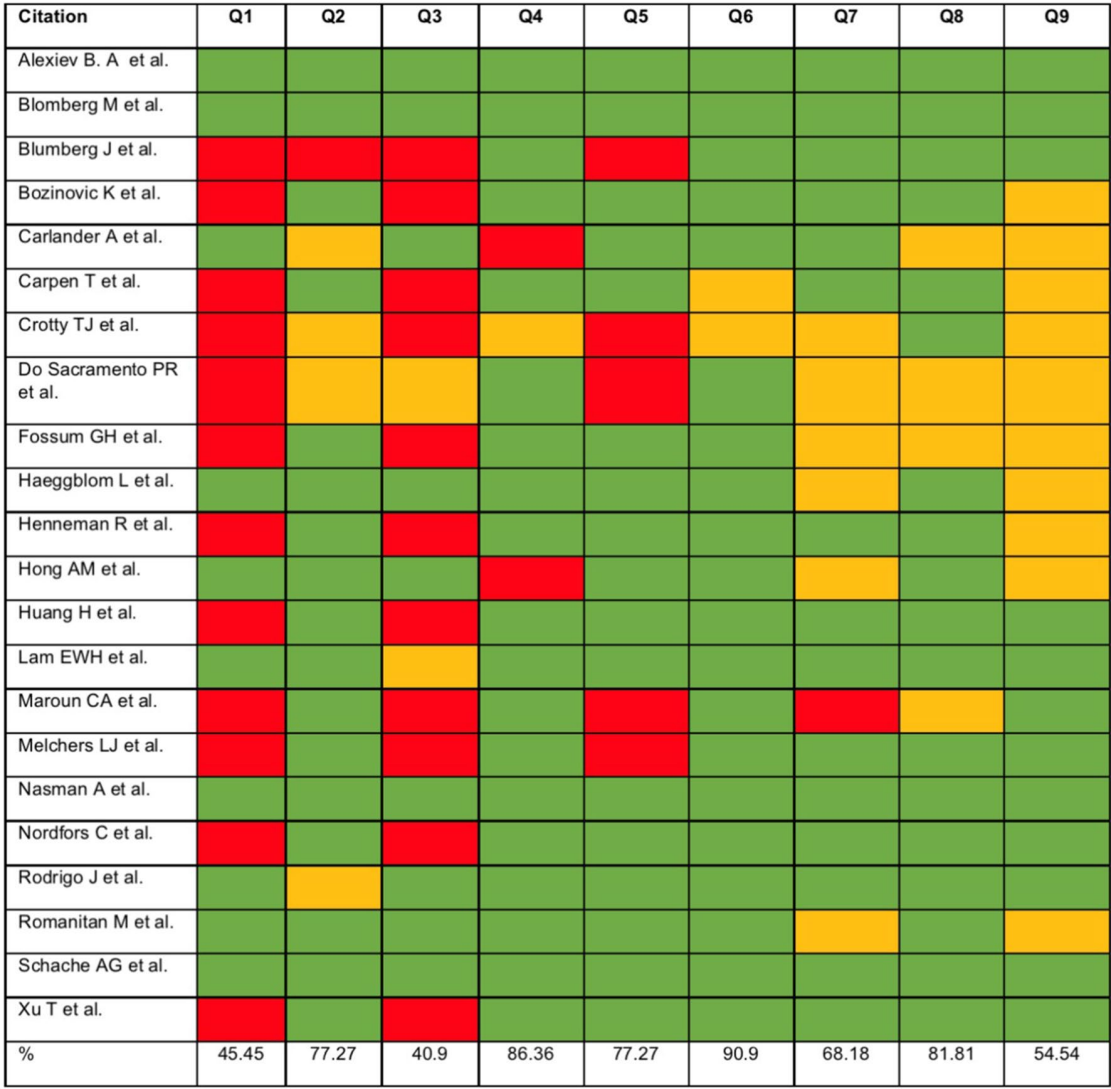
Quality appraisal of included studies

Results from the JBI Appraisal tool ascertained the quality of the included studies and was tabulated (Fig. 2). The green cells represent an answer of ‘yes’ by both of the reviewers for the respective question, red cells represent an answer of ‘no’ by both reviewers for the question, and yellow cells represent ‘unclear’. The ‘%’ column represents the percentage of agreement between the two reviewers for each question. Questions 1 and 3 pertained to the sample size of each study, and substantial disagreement between reviewers was observed for these questions (45.45% and 40.9% agreement respectively), with a total inter-reviewer agreement of 78.6% (Online Appendix 5).

## Pooled Global Prevalence

**Fig. 3:**
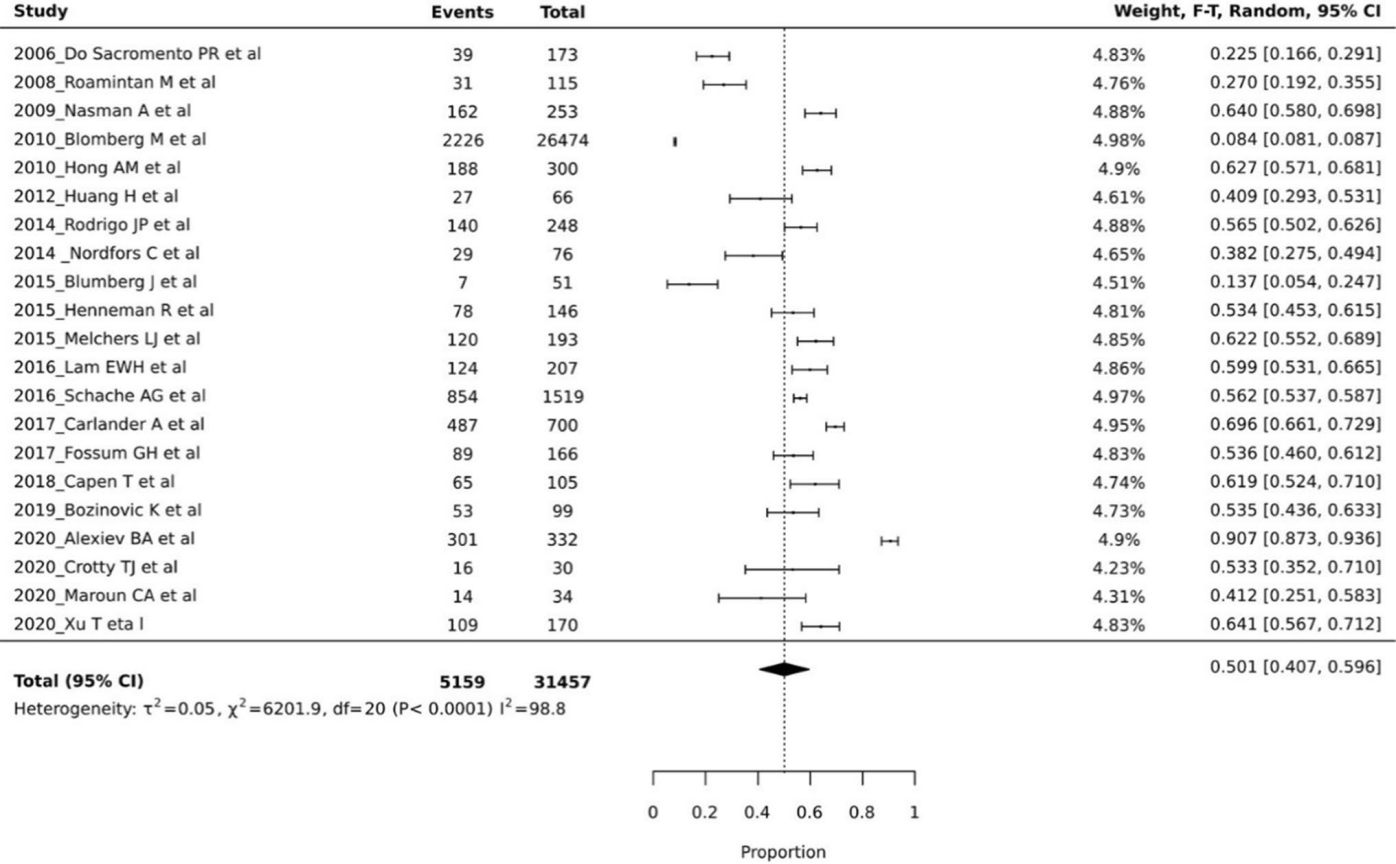
Plot of tonsillar cancer prevalence reported by all included 22 studies

**Fig. 4:**
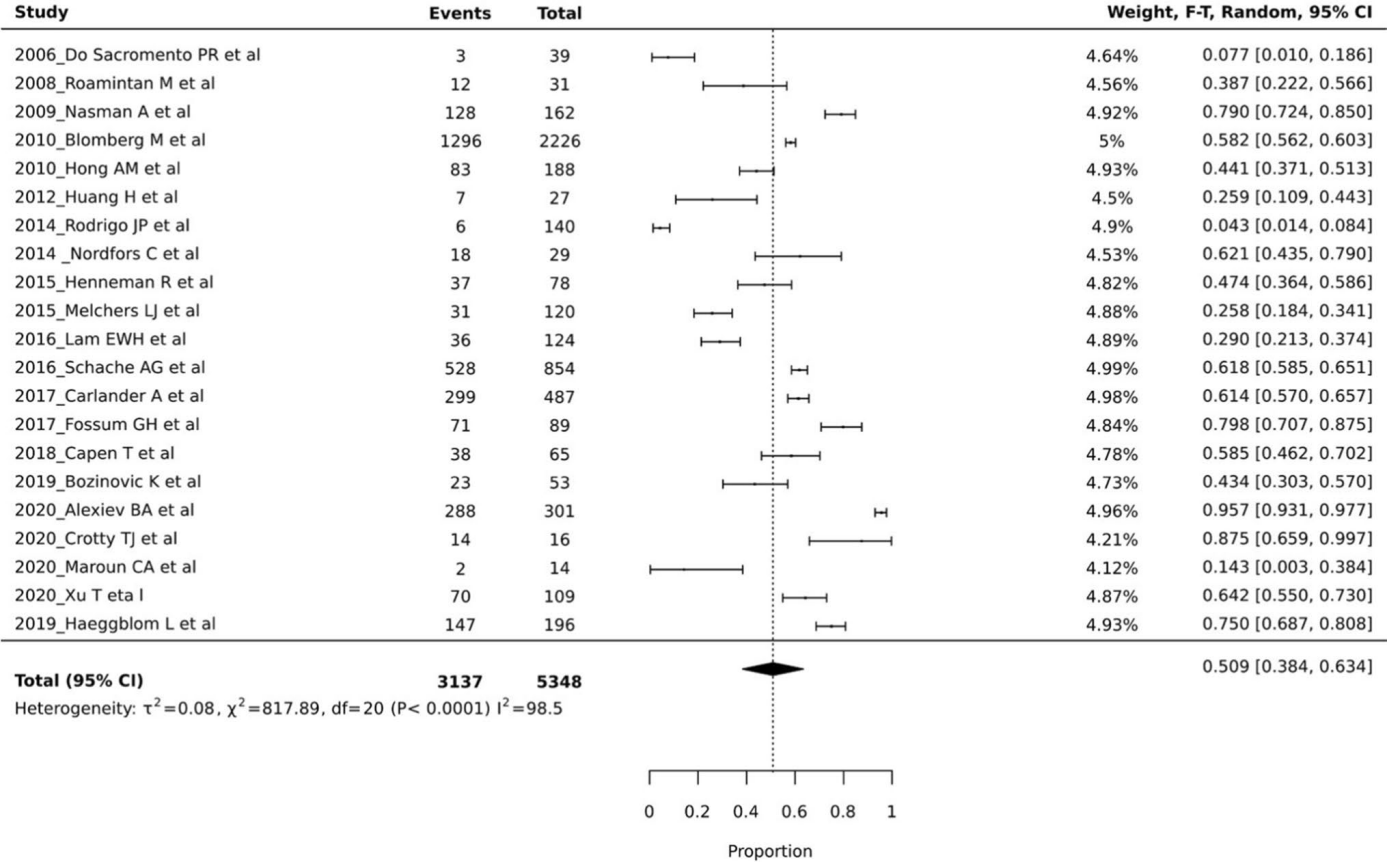
Plot of HPV (+) tonsillar cancer prevalence reported by all included 22 Studies

The prevalence of tonsillar cancers, within the included group of head and neck cancers, ranged from 8.4 to 90.7% (Fig. 2). The pooled prevalence was 50.1% (95% CI 40.7–59.6%), with the heterogeneity between the studies being significantly high (*I*^2^ = 98.8, *p* < 0.0001) (Fig. 3). The prevalence of HPV related tonsillar cancer as per the included studies ranged from 4.3 to 95.7% (Fig. 3). This pooled prevalence was 50.9% (95% CI 38.4–63.4%), again with the heterogeneity levels between the studies being significantly high (I^2^ = 98.5, *p* < 0.0001) (Fig. 4). Due to the lack of adequate desired data, estimation of the prevalence for both tonsillar cancer and HPV (+) tonsillar cancer on the basis of sex, age, ethnicity and continent was not performed.

## Discussion

Human papillomavirus infections are prevalent across the world, and its associated cancer incidence rates continue to rise [1]. Recently, HPV infections have attributed to an increase in OPSCC and TC incidence, making it the sixth most common cancer globally [1, 3]. Studies have reported that more than 90% of the cancers affecting the oropharyngeal region (primarily back of throat and tonsils) are caused by oropharyngeal HPV infections [3]. Despite the clear association, very limited literature has been published to support the molecular mechanisms involved. It is postulated that the build-up of HPV within the lymphatic tissue of the tonsils could precipitate the risk of carcinoma development [38]. One of the most common surgical procedures in the field of otorhinolaryngology are tonsillectomies [39]. Studies have reported that the individual risk of developing tonsillar cancer is decreased after a tonsillectomy [1, 11, 38, 39]. A tonsillectomy characterizes a reduction in the tissue vulnerable to HPV infections, therefore, a reduced likelihood of malignant alterations. Previous research highlights a significant decrease in the incidence of oropharyngeal HPV infections among patients with a history of a tonsillectomy [40].

This systematic review reports a pooled prevalence of tonsillar cancer amongst group of head and neck cancers (50.1%) and HPV (+) related tonsillar cancers (50.9%). Considerable heterogeneity was reported amongst the included studies (I^2^ = 98.8, *p* < 0.0001; I^2^ = 98.5, *p* < 0.0001), which could be explained by the variability in study designs and population characteristics like, sample size, geographical locations, HPV prevalence, methodology for HPV detection and duration of the studies. To address the high levels of heterogeneity, subgroup analyses (on the basis of sample size, continent, sex and age) were intended to be performed, however due to lack of data consistency these analyses could not be performed. Additionally, 14 out of 22 studies were from Europe, thus questioning the external validity of the results. This indicates towards the need for more research and targeted studies from other continents, before more definitive conclusions can be drawn on the prevalence or correlation between HPV infections and tonsillar cancer.

Differences in the prevalence of HPV infections and their associated cancer incidence across different countries, could be due to a wide range of biological factors as well as individual ‘host’ immunogenetic factors affecting the behaviour of different subtypes of HPV [16]. Additionally, other factors that impact the incidence of HPV infection include nutritional deficiencies which could lead to impaired cellular immunity, other infections or diseases, inflammatory conditions, genetic predispositions, and lifestyle habits [16]. In some regions of the world, the prevalence of HPV is quite low, leading to variation in the geographic trends of OPSCC/TC prevalence. Different socio-cultural practices, vaccination rates, and education especially regarding safe sexual practices are also recognised critical factors, affecting the spread of HPV infections [37]. Extensive and prolonged alcohol and tobacco consumption habits exhibit a stronger causal relationship with HPV-unrelated cancer [41, 42]. In the context of HPV related carcinomas, the removal of tonsillar tissue in smokers may perpetuate a depressed local immune response creating a immunosuppressed environment [43].

A tonsillectomy procedure is likely to impact the malignant transformation potential for the palatine lymphoid tissue which is considered the most susceptible to carcinogenic factors [11]. The consideration of these procedures in a prophylactic capacity is inspired by successful comparable models like mastectomies [44] and oophorectomies [45] in vulnerable populations. Research findings from Fakhry et al. [11] report a 60% decrease in development of TC when palatine lymphoid tissue was removed, but did not seem to impact the malignant potential of other head and neck sites or oropharyngeal subsites.

Instead of the pre-emptive tonsillectomies, studies endorse primary prevention by increasing the uptake of vaccinations [1, 11, 38], similar to vaccination strategies for preventing cervix uteri carcinomas.

A limitation of the current review was the inability to assess the impact of HPV vaccinations. HPV vaccination status could provide an insight to the adequacy of the vaccine in prevention of oropharyngeal HPV infections within the oropharyngeal/tonsillar transitional epithelium.

## Conclusion

Hence, further emphasising our point for the need of more representative, specific research on HPV related OPSCC prevalence per region and continent, to allow for a better evaluation of any globally burden. We theorise, that these factors could have all contributed or caused the high heterogeneity seen in both pooled global prevalence’s.

### Supplementary Information

Below is the link to the electronic supplementary material.

PRISMA statement (DOCX 62 KB)

Logic grid (DOCX 19 KB)

JBI Appraisal tool (DOCX 27 KB)

Appraisal results (DOCX 82 KB)

Inter-reviewer reliability (DOCX 15 KB)
