# Supplementary material for: Association of Human Papillomavirus Infection with Tonsillar Cancers: A Systematic Review
Source: Indian J Otolaryngol Head Neck Surg. 2023 Aug 29;76(1):268–76. doi: 10.1007/s12070-023-04140-2 (PMC10908725; doi:10.1007/s12070-023-04140-2)
Supplement: Supplementary file 1 — PRISMA statement (DOCX 62 KB) [file 12070_2023_4140_MOESM1_ESM.docx]

**Identification of studies via other methods**

**Identification of studies via databases and registers**

Records identified from:

Websites (n = 0)

Organisations (n = 0)

Citation searching (n = 14)

Records removed *before screening*:

Duplicate records removed (n =1226)

Records identified from:

Databases (n =2734)

*Pubmed (n = 745)*

*Scopus (n = 320)*

*Embase (n = 543)*

*Web of Science (n = 1126)*

**Identification**

Records screened

(n = 1508)

Records excluded**

(n = 1065)

Reports not retrieved

(n = 3)

Reports sought for retrieval

(n = 11)

Reports sought for retrieval

(n = 210)

Reports not retrieved

(n = 50)

**Screening**

Reports excluded:

Ineligible context (n = 28)

Ineligible participant characteristics (n = 42)

Ineligible outcomes (n = 34)

Abstract only (n = 16)

Ineligible condition (n = 14)

Ineligible study design (n = 4)

Reports assessed for eligibility

(n = 11)

Reports excluded:

Ineligible study design (n = 7)

Ineligible condition (n = 2)

Ineligible participant characteristics (n = 2)

Reports assessed for eligibility

(n = 160)

Studies included in review

(n = 22)

**Included**

*From:*  Page MJ, McKenzie JE, Bossuyt PM, Boutron I, Hoffmann TC, Mulrow CD, et al. The PRISMA 2020 statement: an updated guideline for reporting systematic reviews. BMJ 2021;372:n71. doi: 10.1136/bmj.n71. For more information, visit: <http://www.prisma-statement.org/>
