# Supplementary material for: Association of Human Papillomavirus Infection with Tonsillar Cancers: A Systematic Review
Source: Indian J Otolaryngol Head Neck Surg. 2023 Aug 29;76(1):268–76. doi: 10.1007/s12070-023-04140-2 (PMC10908725; doi:10.1007/s12070-023-04140-2)
Supplement: Supplementary file 2 — Logic grid (DOCX 19 KB) [file 12070_2023_4140_MOESM2_ESM.docx]

**Appendix 2: Logic Grid Search**

23^rd^ December, 2021

Logic Grid: **Pubmed: 745**

HPVAND TONSILLAR CANCER

| HPV | Tonsillar Cancer |
| --- | --- |
| HPV[Text Word] OR "papillomaviridae"[MeSH Terms] OR “human papillomavirus”[MeSH] OR “human papillomavirus”[Text Word] OR "human papillomavirus 16"[MeSH Terms] OR human papillomavirus 16 [Text Word] OR "human papillomavirus 18" [MeSH Terms] OR human papillomavirus 18 [Text Word] OR papillomavirus[Text Word] | ("Tonsils"[Text Word] OR "tonsil*"[All Fields]) AND ("cancer"[Text Word] OR "carcinoma"[Text Word] OR "Malignant"[Text Word]) |

Logic Grid: **EMBASE: 543**

| HPV | Tonsillar Cancer |
| --- | --- |
| ('human papillomavirus type 16':ti,ab,kw OR 'human papillomavirus type 18':ti,ab,kw OR 'wart virus':ti,ab,kw OR 'oral human papillomavirus infection':ti,ab,kw OR 'hpv':ti,ab,kw) | ‘tonsil’:ti,ab,kw OR 'tonsils':ti,ab,kw OR ‘tonsillar cancer' OR ‘tonsillar malignan*’ OR ‘tonsillar carcinoma’ |

Logic Grid **SCOPUS: 320**

| HPV | Tonsillar cancer |
| --- | --- |
| TITLE-ABS-KEY (HPV) OR TITLE-ABS-KEY (papillomaviridae) OR TITLE-ABS-KEY (human papillomavirus) OR TITLE-ABS-KEY (human papillomavirus type 16) OR TITLE-ABS-KEY (human papillomavirus type 18) | TITLE-ABS-KEY ( tonsil and cancer )  OR  TITLE-ABS-KEY ( tonsil  AND malignant )  OR  TITLE-ABS-KEY ( tonsillar carcinoma ) |

**Web of Science: 1126**

| HPV | Tonsillar cancer |
| --- | --- |
| (HPV OR human papillomavirus OR human papillomavirus type 16 OR human papillomavirus type 18 OR Oral HPV OR wart virus) | (‘tonsil’ OR 'tonsils' OR ‘tonsillar cancer' OR ‘tonsillar malignan*’ OR ‘tonsillar carcinoma’) |
