# Supplementary material for: Association of Human Papillomavirus Infection with Tonsillar Cancers: A Systematic Review
Source: Indian J Otolaryngol Head Neck Surg. 2023 Aug 29;76(1):268–76. doi: 10.1007/s12070-023-04140-2 (PMC10908725; doi:10.1007/s12070-023-04140-2)
Supplement: Supplementary file 3 — JBI Appraisal tool (DOCX 27 KB) [file 12070_2023_4140_MOESM3_ESM.docx]

Appendix 4: Appraisal Questions

|  | Yes | No | Unclear | Not applicable |
| --- | --- | --- | --- | --- |
| 1. Was the sample frame appropriate to address the target population? | □ | □ | □ | □ |
| 1. Were study participants sampled in an appropriate way? | □ | □ | □ | □ |
| 1. Was the sample size adequate? | □ | □ | □ | □ |
| 1. Were the study subjects and the setting described in detail? | □ | □ | □ | □ |
| 1. Was the data analysis conducted with sufficient coverage of the identified sample? | □ | □ | □ | □ |
| 1. Were valid methods used for the identification of the condition? | □ | □ | □ | □ |
| 1. Was the condition measured in a standard, reliable way for all participants? | □ | □ | □ | □ |
| 1. Was there appropriate statistical analysis? | □ | □ | □ | □ |
| 1. Was the response rate adequate, and if not, was the low response rate managed appropriately? | □ | □ | □ | □ |

Overall appraisal: Include □ Exclude □ Seek further info □

Comments (Including reason for exclusion)

________________________________________________________________________________________________________________________________________________________________________________________________________________________________________________________________________________________________

Reference

1. Whiting P, Rutjes AWS, Reitsma JB, Bossuyt PMM, Kleijnen J. The development of QUADAS: a tool for the quality assessment of studies of diagnostic accuracy included in systematic reviews. BMC Medical Research Methodology. 2003;3:25 doi:10.1186/1471-2288-3-25.
