# Supplementary material for: Association of Human Papillomavirus Infection with Tonsillar Cancers: A Systematic Review
Source: Indian J Otolaryngol Head Neck Surg. 2023 Aug 29;76(1):268–76. doi: 10.1007/s12070-023-04140-2 (PMC10908725; doi:10.1007/s12070-023-04140-2)
Supplement: Supplementary file 4 — Appraisal results (DOCX 82 KB) [file 12070_2023_4140_MOESM4_ESM.docx]

Appendix 5: Critical Appraisal Results

#### Table: Prevalence Study

| **Citation** | **Q1** | **Q2** | **Q3** | **Q4** | **Q5** | **Q6** | **Q7** | **Q8** | **Q9** |
| --- | --- | --- | --- | --- | --- | --- | --- | --- | --- |
| Allareddy V, Nalliah RP, Haque M, Johnson H, Rampa S, Lee MK. 2014. | Y | Y | Y | Y | Y | Y | Y | U | N/A |
| dos Santos BF, Dabbagh B. 2020. | Y | Y | Y | Y | Y | Y | Y | Y | N/A |
| Jung CP, Tsai AI, Chen CM. 2016. | Y | Y | Y | Y | Y | U | Y | U | Y |
| Oliva MG, Kenny DJ, Ratnapalan S. 2008. | Y | Y | Y | U | Y | Y | Y | U | Y |
| Zeng Y, Sheller B, Milgrom P. 1994. | Y | Y | Y | Y | Y | Y | Y | U | Y |
| Lewis C, Lynch H, Johnston B. 2003. | Y | Y | Y | Y | Y | Y | Y | Y | Y |
| Graham DB, Webb MD, Seale NS. 2000. | Y | Y | U | Y | Y | Y | Y | U | Y |
| Ladrillo TE, Hobdell MH, Caviness AC. 2006. | Y | Y | Y | Y | Y | Y | Y | Y | Y |
| Quiñonez C, Gibson D, Jokovic A, Locker D. 2009. | Y | Y | Y | Y | Y | Y | Y | U | Y |
| Wilson S, Smith GA, Preisch J, Casamassimo PS. 1997. | Y | Y | Y | Y | Y | Y | Y | U | Y |
| Fleming P, Gregg TA, Saunders ID. 1991. | Y | Y | Y | U | Y | Y | Y | U | Y |
| Hong L, Ahmed A, McCunniff M, Liu Y, Cai J, Hoff G. 2011. | Y | Y | Y | Y | Y | Y | Y | Y | Y |
| Rowley ST, Sheller B, Williams BJ, Mancl L. 2006. | Y | Y | Y | N | Y | Y | Y | U | Y |
| Majewski RF, Snyder CW, Bernat JE. 1988. | Y | Y | Y | Y | U | Y | Y | U | Y |
| % | 100.0 | 100.0 | 92.85 | 78.57 | 92.85 | 92.85 | 100.0 | 28.57 | 85.71 |
