# Supplementary material for: Association of Human Papillomavirus Infection with Tonsillar Cancers: A Systematic Review
Source: Indian J Otolaryngol Head Neck Surg. 2023 Aug 29;76(1):268–76. doi: 10.1007/s12070-023-04140-2 (PMC10908725; doi:10.1007/s12070-023-04140-2)
Supplement: Supplementary file 5 — Inter-reviewer reliability (DOCX 15 KB) [file 12070_2023_4140_MOESM5_ESM.docx]

# **Appendix 5: Inter-reviewer reliability for critical appraisal**

| Study | Number of questions in agreement | Number of questions in disagreement | Score |
| --- | --- | --- | --- |
| *Allareddy et al. 2014* | 7 | 2 | 7 |
| *dos Santos and Dabbagh 2020* | 8 | 1 | 8 |
| *Jung et al. 2016* | 6 | 3 | 6 |
| *Oliva et al. 2008* | 6 | 3 | 6 |
| *Zeng et al. 1994* | 7 | 2 | 7 |
| *Lewis et al. 2003* | 9 | 0 | 9 |
| *Graham et al. 2000* | 7 | 2 | 7 |
| *Ladrillo et al. 2006* | 9 | 0 | 9 |
| *Quinonez et al. 2009* | 8 | 1 | 8 |
| *Wilson et al. 1997* | 8 | 1 | 8 |
| *Fleming et al. 1991* | 6 | 3 | 6 |
| *Hong et al. 2011* | 7 | 2 | 7 |
| *Rowley et al. 2006* | 4 | 5 | 4 |
| *Majewski et al. 1988* | 8 | 1 | 8 |
| **Mean** |  |  | **7.14** |
